# Daily supplementation with the Lab4P probiotic consortium induces significant weight loss in overweight adults

**DOI:** 10.1038/s41598-020-78285-3

**Published:** 2021-01-06

**Authors:** D. R. Michael, T. S. Davies, A. A. Jack, G. Masetti, J. R. Marchesi, D. Wang, B. H. Mullish, S. F. Plummer

**Affiliations:** 1 Cultech Limited, Unit 2 Christchurch Road, Baglan Industrial Park, Port Talbot, UK; 2 School of Biosciences, Cardiff University, Cardiff, UK; 3 Division of Digestive Diseases, Department of Metabolism, Digestion and Reproduction, Faculty of Medicine, Imperial College London, London, UK; 4 Department of Clinical Sciences, Liverpool School of Tropical Medicine, Liverpool, UK; 5 Present Address: Department of Cellular Computational and Integrative Biology, University of Trento, Povo, Italy

**Keywords:** Nutrition, Risk factors, Metabolic disorders

## Abstract

This 9-month randomised, parallel, double-blind, single-centre, placebo-controlled study (PROBE, ISRCTN18030882) assessed the impact of probiotic supplementation on bodyweight. Seventy overweight Bulgarian participants aged 45–65 years with BMI 25–29.9 kg/m^2^ received a daily dose of the Lab4P probiotic comprising lactobacilli and bifidobacteria (50 billion cfu/day). Participants maintained their normal diet and lifestyle over the duration of the study. The primary outcome was change from baseline in body weight and secondary outcomes included changes in waist circumference, hip circumference and blood pressure. A significant between group decrease in body weight (3.16 kg, 95% CI 3.94, 2.38, *p* < 0.0001) was detected favouring the probiotic group. Supplementation also resulted in significant between group decreases in waist circumference (2.58 cm, 95% CI 3.23, 1.94, *p* < 0.0001) and hip circumference (2.66 cm, 95% CI 3.28, 2.05, *p* < 0.0001) but no changes in blood pressure were observed. These findings support the outcomes of a previous shorter-term Lab4P intervention study in overweight and obese participants (PROMAGEN, ISRCTN12562026). We conclude that Lab4P has consistent weight modulation capability in free-living overweight adults.

## Introduction

The gastro-intestinal (GI) microbiota is a contributor to human health and has been implicated in many metabolic processes including the digestion and absorption of nutrients and the fermentation of undigested carbohydrates into short chain fatty acids and other metabolites^1^. The link between dysbiosis and obesity is well recognised but nearly 40% of the adults worldwide are categorised as overweight^2^ and this population gains on average 0.2–1.0 kg/year^3^. In addition to progressive weight gain, the aging process itself is characterised by changes in the gut microbiota and the development of a chronic inflammatory status^4^ which is linked to weight associated comorbidities such as diabetes, cardiovascular disease and cancer^5,6^. Consequently, attention is being drawn to the potential for modulation of these changes as an additional approach in the battle against obesity.

The World Health Organisation (WHO) defines probiotic bacteria as ‘live microorganisms that, when administered in adequate amounts confer a health benefit on the host’^7^, and there is accumulating evidence supporting their impact on the gut microbiota resulting in beneficial effects^8–10^. Recent meta-analyses report that body weight reductions have been achieved in short-term probiotic intervention studies (averaging 2 months) in overweight/obese particpants^11–13^. It is considered that a 3% reduction in bodyweight over a 6–12 month period represents successful weight loss^14^ whilst current clinical guidelines state that a 5% reduction is required to illicit significant improvements in health^15^.

In an exploratory placebo-controlled study (PROMAGEN, ISRCTN12562026) the impact of supplementation with the Lab4P probiotic consortium in overweight and obese adults with no dietary or lifestyle restrictions resulted in a significant reduction in body weight (1.5%) over a 6-month period together with improvements in quality-of-life and reductions in the incidence of upper respiratory tract infections^16^. Stratification of the study population indicated greater weight losses in older participants (2% compared to ≤ 1.2% in younger participants) and with 1.9% weight reduction in overweight participants compared to 1.2% in obese participants^16^. The design of the current study was based on the findings from the PROMAGEN study. This study’s aim was to repeat the assessment of the PROMAGEN probiotic supplementation specifically in overweight, older participants, measuring body weight (BW), waist circumference (WC), hip circumference (HC) and blood pressure (BP).

## Methods

### Study approval

This study was a single-centre, double-blind, randomised and placebo-controlled superiority study with equal allocation of participants between two parallel study groups. The study was approved by the Ethical Committee of Comac Medical, Sofia, Bulgaria (Reference: #168/08.05.2019), registered with IRSCTN (ISRCTN18030882, Registration date: 27.11.2019) and was conducted in accordance with the principles of the Declaration of Helsinki.

### Recruitment, participants and randomisation

The trial was performed by Comac Medical Ltd and healthy Bulgarian adults (aged 45–65; BMI 25–29.9 kg/m^2^; WC > 89 cm for women and > 100 cm for men) providing written informed consent were recruited at a trial facility (Sofia, Bulgaria) between 17/05/2019 and 22/05/2019.

Sample size calculations were based on the body weight changes observed in a subgroup of PROMAGEN participants (aged 45–65, BMI 25–29.9 kg/m^2^) and 31 participants per group were required to detected a 1.54 kg reduction (standard deviation of 2.01 kg) using a Type I error of 0.05 and a power of 85%. Thirty-five participants per group were selected to account for potential drop-outs.

Participants were excluded if they were undergoing immunodeficiency/immunosuppressive therapy; pregnant or planning pregnancy; had history of ischemic heart disease, heart failure, prolonged QTc interval, rhythm and conduction disorders—absolute arrhythmia, ventricular extrasystole, atrioventricular block or any other cardiovascular disease deemed by the investigator as a risk for the participation in the study; had severe systemic disease (cancer, dementia, advanced organ failure); had experienced significant weight loss in the last 3 months that could not be explained by a dietary regimen or increased physical activity; or if they had received any statin therapy in the 6 months prior to the study period.

All participants were sequentially allocated to one of two arms of the study in a 1:1 ratio according to a computer-generated random sequence (block-size of four using SAS PROC PLAN (SAS version 9.4)). An independent statistician provided the randomisation code and the trial product was randomised before arrival at the trial site. The allocation sequence was not made available to researchers until all databases were completed and locked (the allocation sequence was held at the trial site in tamper-proof sealed envelopes in case of emergency).

### Study design and intervention

The randomised participants received one capsule per day of either the active Lab4P product or a matching placebo for 9 months (270 days). Participants were advised to take the intervention with food (with or without a cool drink) at any time of day within no less than 2 h of any antibiotic intake. All participants were required to avoid the consumption of any probiotics and maintain their normal diet and lifestyle throughout the study.

The active product (Lab4P) was identical to that used in PROMAGEN^16^ and comprised *Lactobacillus acidophilus* CUL60 (NCIMB 30157), *Lactobacillus acidophilus* CUL21 (NCIMB 30156), *Lactobacillus plantarum* CUL66 (NCIMB 30280), *Bifidobacterium bifidum* CUL20 (NCIMB 30153) and *Bifidobacterium animalis* subsp. *lactis* CUL34 (NCIMB 30172) at a total of 5 × 10^10^ colony forming units (cfu) per capsule. The placebo capsules comprised microcrystalline cellulose and were identical in appearance to the active product. All capsules were prepared by Cultech Ltd, Port Talbot, UK and were provided in induction-sealed pots. The trial products were stored between 4 and 8 °C at the trial site and participants were required to refrigerate the intervention throughout the study. Unused capsules were collected for compliance monitoring.

### Study outcomes

The primary outcome was change from baseline in body weight (BW). Secondary outcomes were changes from baseline in waist circumference (WC), hip circumference (HC) and blood pressure (BP). Other secondary outcomes are not included in this report.

### Data and sample collection

Participants were required to attend the study centre on four occasions: Baseline (day 0), 3 months (day 90 ± 3), 6 months (day 180 ± 3) and 9 months (day 270 ± 3). The height of participants was recorded at baseline and BW, WC, HC and BP were measured at all visits. Pots of 93 capsules were provided to participants at the baseline, 3 and 6 month visits and unused capsules collected at the 3, 6 and 9 month visits.

### Physiological measurements

All physiological measurements were made as previously described^16^. Briefly, BW was recorded using a calibrated column scale (Seca 709, Hamburg, Germany) after the removal of shoes and jackets; WC was measured 2 fingers below the umbilicus; HC was measured at the level of maximum protrusion; seated BP was measured after 5 min respite using a calibrated blood pressure monitor (Omron, Kyoto, Japan) and height was measured after the removal of shoes. The time at which measurements were taken was standardised for each participant wherever possible.

### Data management and statistical analysis

Data was analysed as previously described^16^. Briefly, analysis of study outcomes was performed on an intention-to-treat basis using a linear mixed model (LMM) that included treatment, time and interaction between treatment and time as fixed effects, baseline measurements as the covariate and subject as the random effect. Differences between treatment groups at each time point with 95% confidence intervals (CI) from a t-test were calculated from the LMM. Covariate adjusted analyses within the LMM framework as described above were performed on all outcomes with age, gender and BMI as covariates. Values of *p* were considered statistically significant when less than 0.05. Continuous variables were summarised using mean ± standard deviation (SD). Data analyses were performed using SAS version 9.4 (SAS Institute Inc., Cary, NC, USA).

## Results

### Recruitment

**Figure 1:**
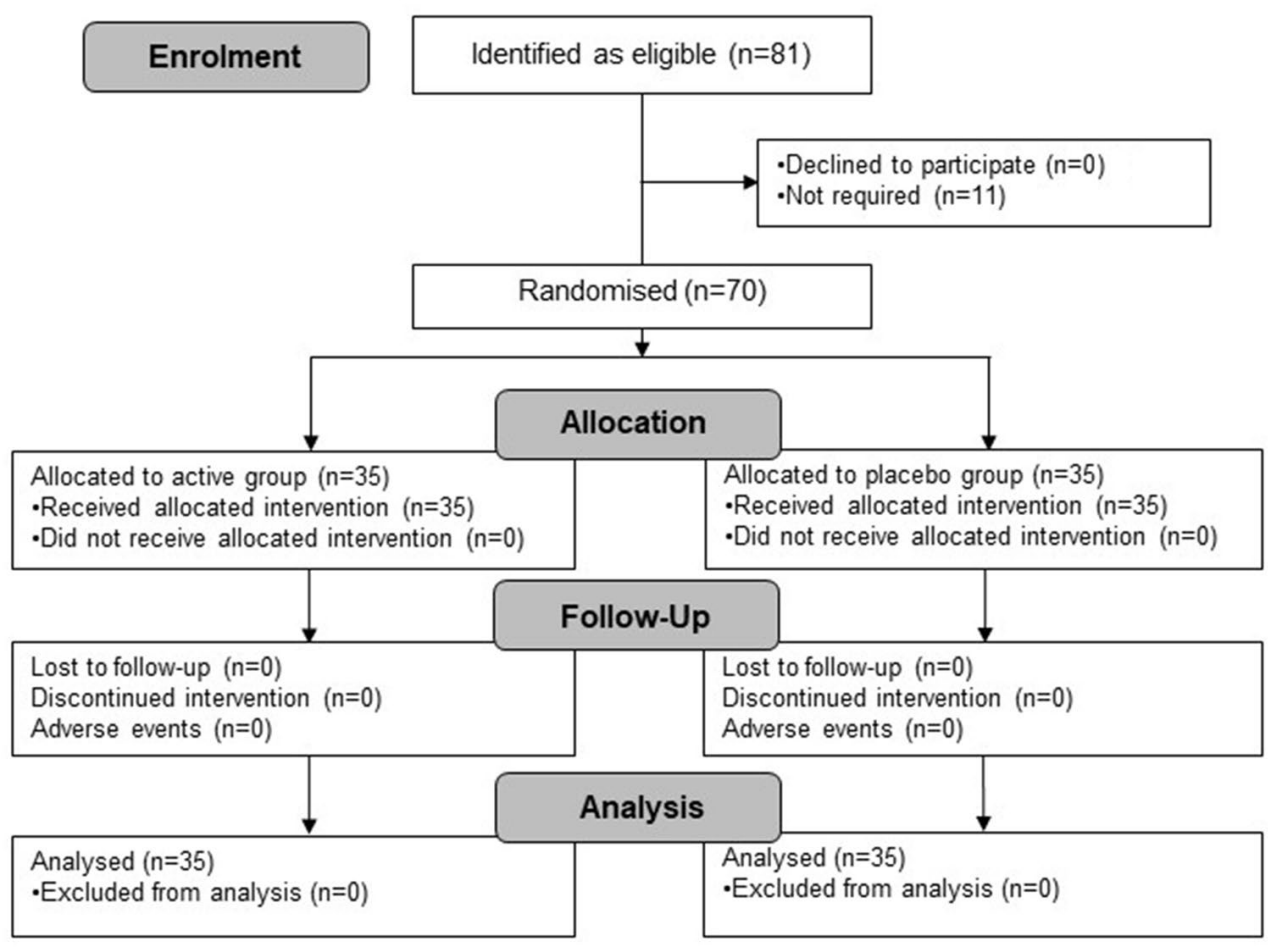
Flow diagram of study.

Eighty-one conforming candidates were identified from a database of healthy participants and 70 of these participants were recruited. The study took place between May 2019 and February 2020 and there were no drop-outs, exclusions or adverse events in either arm of the study (Fig. 1). Compliance to the intervention (as defined by number of returned capsules) exceeded 99% in both arms of the study.

### Physiological measurements

**Figure 2:**
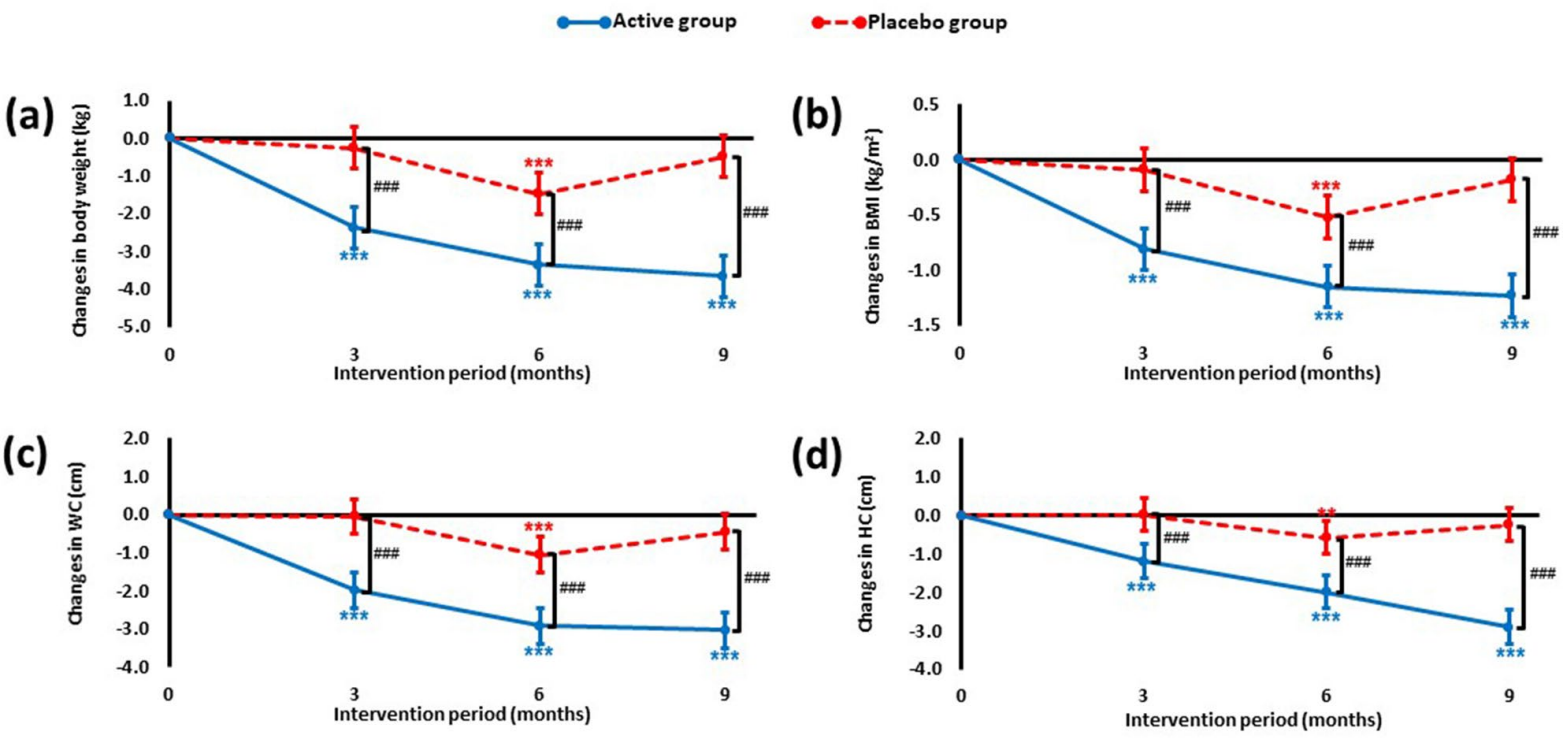
Changes from baseline in (**a**) body weight, (**b**) BMI, (**c**) waist circumference and (**d**) hip circumference over the duration of the intervention period. Data is presented as mean change from baseline (35 participants per group) with 95% CI and *p* values calculated using a LMM. For within group comparisons (vs. baseline): **p ≤ 0.01 and ***p ≤ 0.001. For between group comparisons (active vs. placebo): ^###^*p* ≤ 0.001.

Baseline demographics of the participants are shown in Table 1. Changes in BW, BMI, WC and HC are shown in Fig. 2 (detailed data presented in Supplementary Table S1). Significant between group reductions in BW compared to the placebo were seen at 3 months (− 2.51%, − 2.11 kg, *p* < 0.0001), 6 months (− 2.19%, − 1.88 kg, *p* < 0.0001) and 9 months where weight loss approached 4% (− 3.76%, − 3.16 kg, *p* < 0.0001, Fig. 2a). Weight loss from baseline for the probiotic group was consistent and reached significance at 3 months (− 2.83%, − 2.37 kg, *p* < 0.0001), 6 months (− 4.00%, − 3.35 kg, *p* < 0.0001) and 9 months (− 4.36%, − 3.65 kg, *p* < 0.0001). Significant weight loss was recorded in the placebo group at 6 months (− 1.81%, − 1.47 kg, *p* < 0.0001), but the weight of the participants in the placebo group returned to near baseline by 9 months. Changes in BMI over the course of the study were consistent with weight loss results (Fig. 2b).

**Table 1:** Demographic and baseline characteristics of the PROBE population.

	Active (n = 35)	Placebo (n = 35)
	Mean (SD)	Mean (SD)
**Study demographics**		
Age (years)	52.40 (5.84)	55.26 (5.79)
Males (n (%))	18 (51.4%)	19 (54.3%)
Females (n (%))	17 (48.6%)	16 (45.7%)
**Anthropometry**		
Weight (kg)	83.66 (11.26)	81.16 (11.24)
Height (m)	1.72 (0.10)	1.70 (0.10)
BMI (kg/m^2^)	28.10 (1.55)	27.90 (1.54)
WC (cm)	104.20 (9.60)	106.37 (11.93)
HC (cm)	112.40 (6.65)	111.06 (7.25)
SBP (mmHg)	127.11 (10.45)	129.49 (9.56)
DBP (mmHg)	81.00 (7.04)	84.26 (6.75)
WtHR	0.61 (0.05)	0.63 (0.06)
WC:HC	0.93 (0.09)	0.96 (0.09)
Conicity Index	1.37 (0.10)	1.42 (0.14)

The data represents the mean ± standard deviation (SD) of 35 participants in each group. The number of participants (n) that were male or female in each group are expressed as a percentage of the total group size. *BMI* body mass index, *WC* waist circumference, *HC* hip circumference, *SBP* systolic blood pressure, *DBP* diastolic blood pressure, *WtHR* waist-to-height ratio, *WC:HC* waist-to-hip ratio.

The weight loss was reflected in significant between group reductions in both WC and HC in response to the probiotic at each time point and by 9 months had dropped by 2.48% (− 2.58 cm, *p* < 0.0001, Fig. 2c) for WC and 2.36% (− 2.66 cm, *p* < 0.0001, Fig. 2d) for HC. These changes contributed to significant reductions in the waist-to-height ratio (WtHR) and the conicity index $({\text{WC}}\;({\text{cm}}){/}(0.109\surd {\text{BW}}({\text{kg}}){\text{/Height}}\;({\text{m}})))$^17^ in the probiotic group (Supplementary Table S1). No changes in systolic or diastolic blood pressure were recorded during the study.

### Rates of successful weight loss (SWL) and clinical weight loss (CWL)

**Figure 3:**
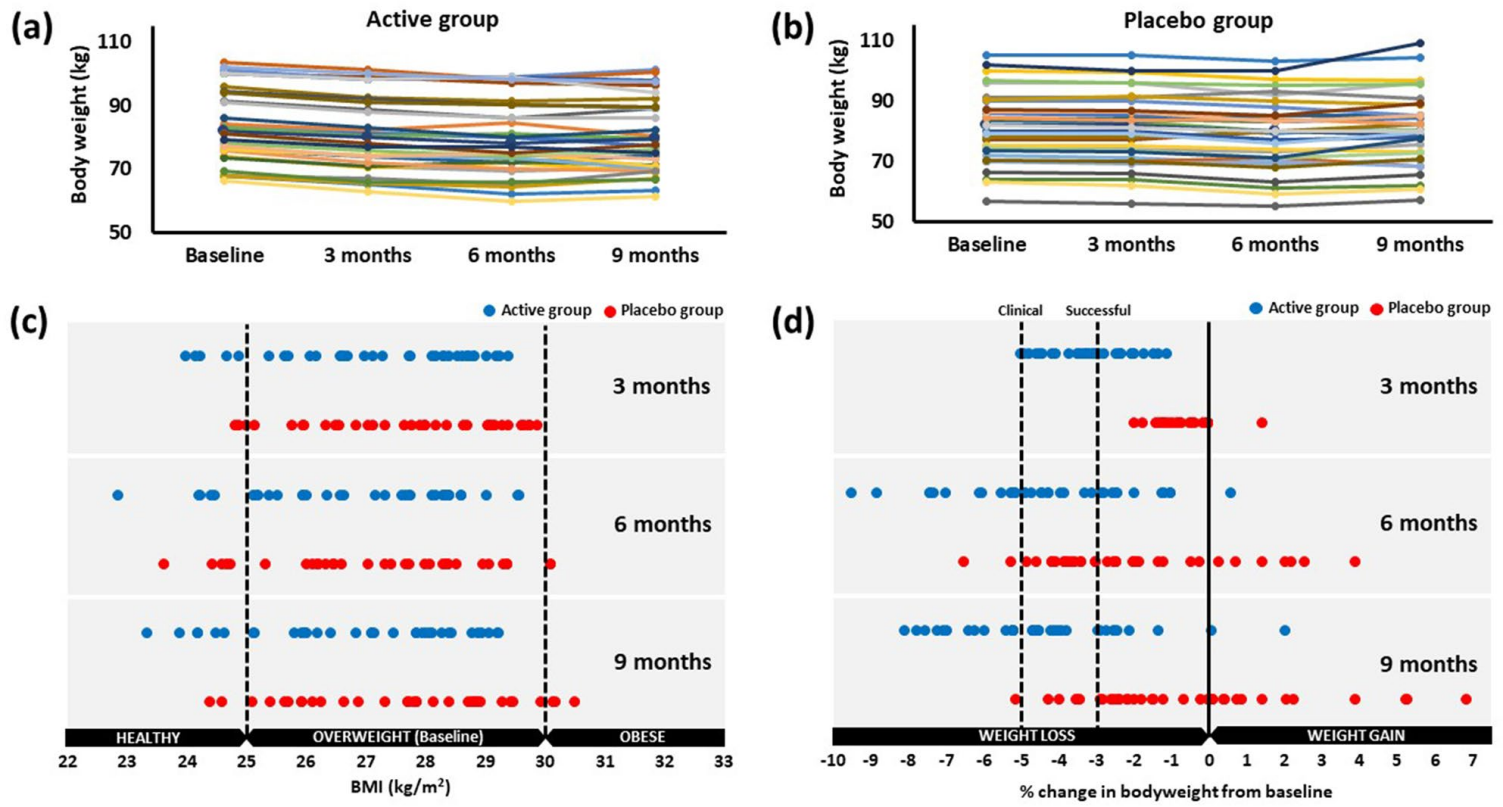
Absolute changes in body weight for each participant in the (**a**) active group or (**b**) placebo group over the duration of the study and the distribution of changes in participant (**c**) BMI and (**d**) percentage change in body weight. Data is presented for 35 participants per group.

Figure 3 clearly indicates consistency of the weight loss in the active group with 33/35 participants achieving weight loss by 9 months (Fig. 3a). In the placebo group there was little weight loss in the first 6 months, and then weight regain at 9 months (Fig. 3b). At the end of the study, six participants from the active group moved from overweight BMI status to healthy BMI status (< 24.9 kg/m^2^) compared with two participants in the placebo group (Fig. 3c). Three participants in the placebo group progressed into the obese BMI status (≥ 30 kg/m^2^) but none in the active group.

**Figure 4:**
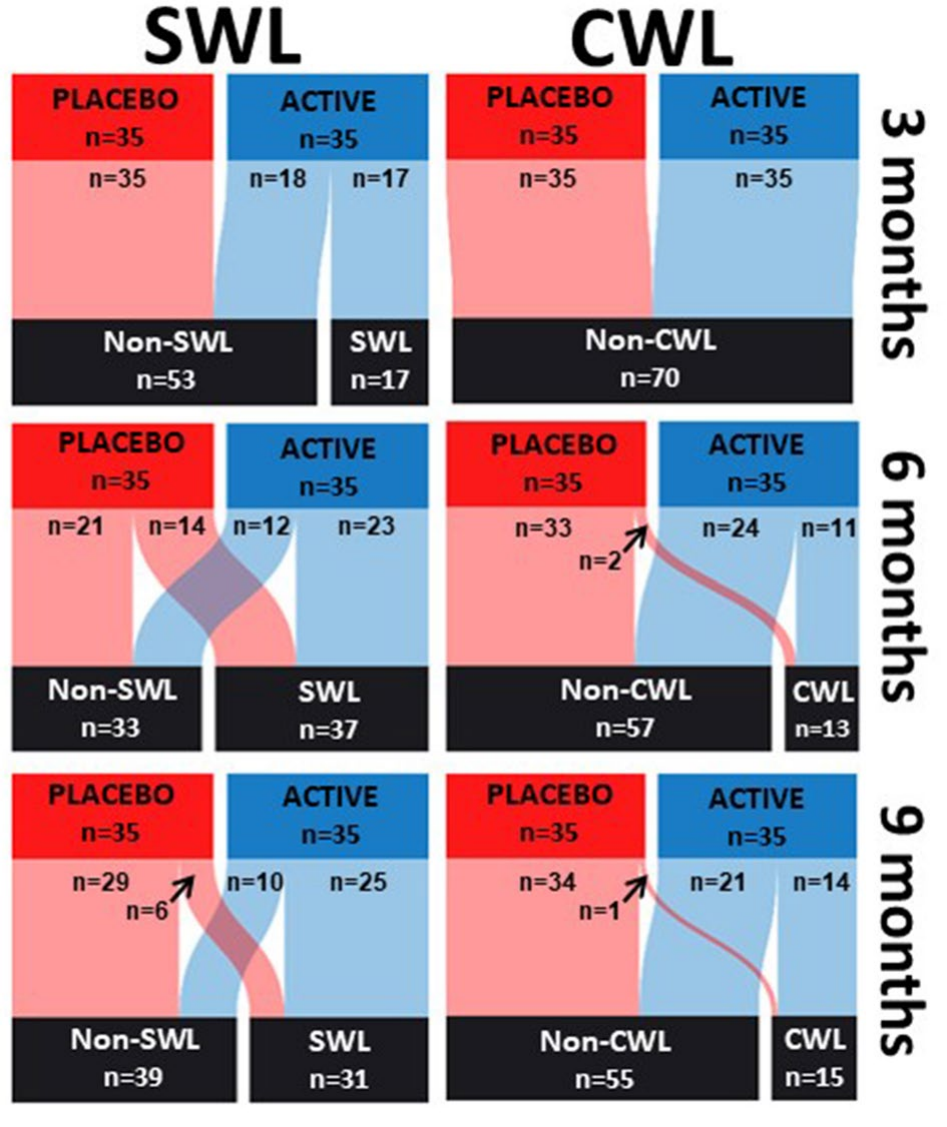
The number of participants achieving successful weight loss and clinical weight loss after 3 months, 6 months and 9 months. Data is presented for 35 participants per group. *SWL* successful weight loss, *CWL* clinical weight loss. Diagram created using sankeyMATIC (http://sankeymatic.com/faq/).

The distribution of percentage weight change can be seen in Fig. 3d. In the early stages of the study all participants lost or maintained weight (except one in the placebo group) with greater percentage weight losses in the active group. A weight loss of ≥ 3% (considered “successful” weight loss by Grembi et al.^14^) was achieved by 17/35 active group participants and 0/35 placebo group participants after 3 months supplementation (49% vs. 0% respectively, *p* < 0.0001, Figs. 3d, 4). By 9 months more than 70% of the active group had achieved 3% weight loss compared with less than 20% of the placebo group (71% vs. 17% respectively, *p* < 0.0001).

In both groups there were instances where weight loss had reached levels that could be considered clinically meaningful (defined as 5% weight loss over a 6–12 month period^15^) and these reductions were more prevalent in the active group compared to the placebo at both 6 months (31% vs. 6%, *p* = 0.0071) and 9 months (40% vs. 3%, *p* = 0.0002).

## Discussion

Daily supplementation of a free-living population of healthy overweight individuals aged 45–65 with the Lab4P probiotic for 9 months resulted in a significant reduction in body weight of 3.8% together with a 2.5% reduction in waist circumference and a 2.4% reduction in hip circumference. No significant changes in blood pressure were detected.

In our previous 6-month exploratory study assessing the impact of the Lab4P probiotic (PROMAGEN) in free-living overweight and obese adults, weight-loss was identified as a significant outcome^16^. At 6 months, significant body weight reductions were seen in the probiotic treated group compared to the placebo (− 1.30 kg, 95% CI − 1.77, − 0.83, *p* < 0.0001) and sub-group analysis of the population revealed the highest weight losses in participants aged 50 or over (2% reduction, *p* = 0.0002) or in the overweight category (1.9% reduction, *p* < 0.0001)^16^. In the meta-analysis of Koutnikova et al., greater levels of probiotic-mediated weight loss were observed in overweight participants compared to the obese^11^. Although much attention is focused on obese individuals, it is estimated that about 70% of adults in England aged between 45 and 65 are classified as overweight^18^ and these people need support to prevent their progression into an obese state due to the anticipated annual weight gain^3^.

PROBE was designed as the follow-up to the PROMAGEN study with the aim of confirming the probiotic-associated weight losses observed in overweight adults^16^ and significant reductions in body weight occurred in the absence of any restrictions imposed on daily diet or lifestyle. Studies demonstrating consistent probiotic-mediated weight loss are extremely rare with existing reports limited to Asian populations and the results of these are variable^19–22^. Numerous meta-analyses have attempted to estimate the overall probiotic weight loss effect from studies with highly variable designs (many with lifestyle/dietary restrictions), interventions (strain and dose) and target populations and have reported reductions in the region of 0.5–1%^11,23^ or no effect^12,24^. We are one of the first groups to demonstrate a consistent weight loss effect across two studies in free-living western populations.

The intervention period in the PROBE study was extended to 9 months to identify if the weight changes observed over the 6-month period in the PROMAGEN study might be on-going. We found that weight loss continued up to 9 months at which point the between group difference in body weight approached 3.2 kg (~ 7 lb). Clinically meaningful levels of weight loss are defined as a 5% reduction in body weight over a 6–12 month period and are associated with measurable metabolic benefits (such as improvements in glycaemic index and plasma lipid levels) and reduced healthcare costs^15,25^. Using ≥ 5% weight loss as a potentially beneficial reference point, 40% of the Lab4P treated group reached this “clinical” target after 9 months supplementation compared to only 3% of participants in the placebo group.

In both the PROBE and PROMAGEN studies, the intervention period spanned Christmas and New Year and weight gains of 0.4–1.0 kg have been observed in the general population over this period^3^. In the PROBE study, the placebo group gained weight during this period (between 6 to 9 months), and this gain counterbalanced the overall weight losses observed in the first 6 months. However, modest weight loss was maintained in the probiotic group through this period. This difference raises the possibility that Lab4P supplementation may help prevent the weight regain observed in the placebo group after weight loss that is a near-ubiquitous problem driven by physiological, psychological and environmental factors^26^. It has been found that more than half of the weight lost by an individual is regained within 2 years of the initial weight loss^27^.

The PROBE study reiterated the observed reduction in waist circumference in the PROMAGEN study and also demonstrated a reduction of hip circumference and the waist/hip ratio. These criteria are used to assess abdominal (central) obesity and elevated waist/hip measurements, and have been linked to the development of cardiovascular disease^28,29^ and dementia^30^.

Numerous mechanisms of action for probiotic-mediated weight loss have been proposed. These include the deconjugation of bile acids by bacterial bile salt hydrolases (BSH) which has been shown to impact on a number of key physiological processes in the host including lipid metabolism^31,32^. Studies in vitro with the Lab4P probiotics indicate that this consortium expresses BSH activity^33,34^ and functionality was confirmed in mouse studies where Lab4P treated C57BL/6 mice had increased faecal levels of deconjugated bile acids that occurred alongside the inhibition of high fat diet induced weight gain^34^. In the PROMAGEN study with adults, an association between the weight loss and high baseline faecal levels of sulphated bile acids was identified, leading us to conclude that host physiology may also be a contributory factor influencing the probiotic associated weight loss^35^.

The strengths of our study include the powered sample size, the 9-month intervention period and the unadjusted lifestyle conditions, that give our findings applicability to the ‘real world’ setting. The limitations of our study include the initial weight loss observed in the placebo group although this did not persist over the entirety of our study and the placebo effect has become a recognised feature of obesity research^36^. The specific BMI range and single geographical isolation of our study population provides uniformity and continuity on one hand but requires further work in both obese and ideal weight participants and in another setting.

In summary, the PROBE intervention study has demonstrated that 9 months Lab4P supplementation at 5 × 10^10^ cfu/day significantly reduced bodyweight, BMI, waist circumference and hip circumference in a free-living overweight population aged 45–65 years old. These results agreed with the outcomes a previous exploratory study and highlight the potential for a role for probiotic supplementation within the weight management arena.

## Supplementary Information

Supplementary Table S1.

## Data Availability

The datasets generated during and/or analysed during the current study are available from the corresponding author on reasonable request.
