# Supplementary material for: Daily supplementation with the Lab4P probiotic consortium induces significant weight loss in overweight adults
Source: Sci Rep. 2021 Jan 6;11:5. doi: 10.1038/s41598-020-78285-3 (PMC7788077; doi:10.1038/s41598-020-78285-3)
Supplement: Supplementary file 1 — Supplementary Table S1. [file 41598_2020_78285_MOESM1_ESM.docx]

Daily supplementation with the Lab4P probiotic consortium induces significant weight loss in overweight adults

D. R. Michael, T. S. Davies, A. A. Jack, G. Masetti, J. R. Marchesi, D. Wang, B. H. Mullish and S. F. Plummer

**SUPPLEMENTARY TABLE S1: Changes from baseline in anthropometry and blood pressure**

| **Outcome** | **Group(s)** | **N** | **3 Months** | | |  | **6 Months** | | |  | **9 Months** | | |
| --- | --- | --- | --- | --- | --- | --- | --- | --- | --- | --- | --- | --- | --- |
|  |  |  | **Difference**  **(95% CI)** | **% change** | ***p* value** |  | **Difference**  **(95% CI)** | **% change** | ***p* value** |  | **Difference**  **(95% CI)** | **% change** | ***p* value** |
| **Weight (kg)** | Between | 35/35 | -2.11 (-2.89, -1.33) | -2.51 | <0.0001 |  | -1.88 (-2.66, -1.10) | -2.19 | <0.0001 |  | -3.16 (-3.94, -2.38) | -3.76 | <0.0001 |
|  | Active | 35 | -2.37 (-2.92, -1.82) | -2.83 | <0.0001 |  | -3.35 (-3.90, -2.80) | -4.00 | <0.0001 |  | -3.65 (-4.20, -3.10) | -4.36 | <0.0001 |
|  | Placebo | 35 | -0.26 (-0.81, 0.29) | -0.32 | 0.3511 |  | -1.47 (-2.02, -0.92) | -1.81 | <0.0001 |  | -0.49 (-1.04, 0.06) | -0.60 | 0.0809 |
| **BMI (kg/m^2^)** | Between | 35/35 | -0.71 (-0.98, -0.44) | -2.56 | <0.0001 |  | -0.62 (-0.89, -0.35) | -2.23 | <0.0001 |  | -1.05 (-1.32, -0.78) | -3.73 | <0.0001 |
|  | Active | 35 | -0.81 (-1.00, -0.62) | -2.88 | <0.0001 |  | -1.15 (-1.34, -0.95) | -4.09 | <0.0001 |  | -1.23 (-1.42, -1.04) | -4.38 | <0.0001 |
|  | Placebo | 35 | -0.09 (-0.28, 0.10) | -0.32 | 0.3373 |  | -0.52 (-0.71, -0.33) | -1.86 | <0.0001 |  | -0.18 (-0.37, 0.01) | -0.65 | 0.0628 |
| **WC (cm)** | Between | 35/35 | -1.93 (-2.57, -1.28) | -1.85 | <0.0001 |  | -1.87 (-2.51, -1.23) | -1.82 | <0.0001 |  | -2.58 (-3.23, -1.94) | -2.48 | <0.0001 |
|  | Active | 35 | -1.98 (-2.43, -1.52) | -1.90 | <0.0001 |  | -2.92 (-3.38, -2.47) | -2.80 | <0.0001 |  | -3.03 (-3.49, -2.58) | -2.91 | <0.0001 |
|  | Placebo | 35 | -0.05 (-0.51, 0.40) | -0.05 | 0.8256 |  | -1.05 (-1.51, -0.60) | -0.99 | <0.0001 |  | -0.45 (-0.91, 0.00) | -0.42 | 0.0521 |
| **HC (cm)** | Between | 35/35 | -1.21 (-1.82, -0.46) | -1.08 | 0.0002 |  | -1.41 (-2.02, -0.79) | -1.25 | <0.0001 |  | -2.66 (-3.28, -2.05) | -2.36 | <0.0001 |
|  | Active | 35 | -1.19 (-1.62, -0.75) | -1.06 | <0.0001 |  | -1.99 (-2.42, -1.55) | -1.77 | <0.0001 |  | -2.90 (-3.34, -2.47) | -2.58 | <0.0001 |
|  | Placebo | 35 | 0.02 (-0.42, 0.45) | 0.02 | 0.9358 |  | -0.58 (-1.02, -0.15) | -0.52 | 0.0092 |  | -0.24 (-0.67, 0.20) | -0.22 | 0.2789 |
| **SBP (mmHg)** | Between | 35/35 | 0.50 (-1.12, 2.12) | 0.40 | 0.5407 |  | 0.82 (-0.80, 2.44) | 0.64 | 0.3206 |  | 1.10 (-0.52, 2.72) | 0.86 | 0.1807 |
|  | Active | 35 | 0.62 (-0.52, 1.77) | 0.49 | 0.2830 |  | 1.02 (-0.12, 2.17) | 0.80 | 0.0789 |  | 1.02 (-0.12, 2.17) | 0.80 | 0.0789 |
|  | Placebo | 35 | 0.12 (-1.02, 1.26) | 0.09 | 0.8355 |  | 0.21 (-0.94, 1.35) | 0.16 | 0.7221 |  | -0.08 (-1.22, 1.06) | -0.06 | 0.8903 |
| **DBP (mmHg)** | Between | 35/35 | 0.21 (-1.38, 1.81) | 0.26 | 0.7913 |  | 0.07 (-1.53, 1.67) | 0.08 | 0.9297 |  | -0.07 (-1.67, 1.53) | -0.13 | 0.9297 |
|  | Active | 35 | -0.02 (-1.14, 1.10) | -0.02 | 0.9698 |  | 0.01 (-1.11, 1.12) | 0.01 | 0.9899 |  | -0.74 (-1.85, 0.38) | -0.91 | 0.1952 |
|  | Placebo | 35 | -0.24 (-1.35, 0.88) | -0.28 | 0.6773 |  | -0.06 (-1.18, 1.05) | -0.07 | 0.9096 |  | -0.66 (-1.78, 0.45) | -0.78 | 0.2419 |
| WtHR | Between | 35/35 | -0.011 (-0.015, -0.008) | -1.97 | <0.0001 |  | -0.011 (-0.015, -0.007) | -1.83 | <0.0001 |  | -0.015 (-0.019, -0.011) | -2.47 | <0.0001 |
|  | Active | 35 | -0.012 (-0.014, -0.009) | -1.97 | <0.0001 |  | -0.017 (-0.020, -0.014) | -2.79 | <0.0001 |  | -0.018 (-0.020, -0.015) | -2.95 | <0.0001 |
|  | Placebo | 35 | -0.000 (-0.003, 0.002) | 0.00 | 0.8408 |  | -0.006 (-0.009, -0.004) | -0.95 | <0.0001 |  | -0.003 (-0.005, 0.000) | -0.48 | 0.0542 |
| **WC:HC** | Between | 35/35 | -0.007 (-0.011, -0.002) | -0.76 | 0.0028 |  | -0.005 (-0.009, -0.000) | -0.55 | 0.0319 |  | -0.000 (-0.005, 0.004) | -0.11 | 0.9365 |
|  | Active | 35 | -0.008 (-0.011, -0.004) | -0.86 | <0.0001 |  | -0.010 (-0.013, -0.007) | -1.08 | <0.0001 |  | -0.003 (-0.006, 0.001) | -0.32 | 0.1110 |
|  | Placebo | 35 | -0.001 (-0.004, 0.002) | -0.10 | 0.6145 |  | -0.005 (-0.008, -0.002) | -0.52 | 0.0026 |  | -0.002 (-0.005, 0.001) | -0.21 | 0.1384 |
| **Conicity Index** | Between | 35/35 | -0.008 (-0.014, -0.002) | -0.58 | 0.0074 |  | -0.010 (-0.015, -0.004) | -0.66 | 0.0014 |  | -0.008 (-0.014, -0.002) | -0.59 | 0.0079 |
|  | Active | 35 | -0.006 (-0.010, -0.002) | -0.44 | 0.0030 |  | -0.010 (-0.014, -0.006) | -0.73 | <0.0001 |  | -0.010 (-0.014, -0.006) | -0.73 | <0.0001 |
|  | Placebo | 35 | 0.002 (-0.002, 0.006) | 0.14 | 0.4040 |  | -0.001 (-0.005, 0.003) | -0.07 | 0.7313 |  | -0.002 (-0.006, 0.002) | -0.14 | 0.4186 |

Data is presented as mean change (35 participants per group) with 95% confidence intervals (CIs) and *p* values calculated using a linear mixed model (LMM). BMI, body mass index; WC, waist circumference; HC, hip circumference; SBP, systolic blood pressure; DBP, diastolic blood pressure; WtHR, waist-to-height ratio; WC:HC, waist-to-hip ratio.
